# Exercise intensity and physical fitness modulate lipoproteins profile during acute aerobic exercise session

**DOI:** 10.1038/s41598-020-61039-6

**Published:** 2020-03-05

**Authors:** B. M. Antunes, F. E. Rossi, L. M. Oyama, J. C. Rosa-Neto, F. S. Lira

**Affiliations:** 1 Exercise and Immunometabolism Research Group, Post-Graduation Program in Movement Sciences, Department of Physical Education, São Paulo State University (UNESP), Presidente Prudente, SP, Brazil; 2 Universidade Federal de São Paulo, Escola Paulista de Medicina, Departamento de Fisiologia, São Paulo, Brazil; 3 Immunometabolism Research Group, Department of Cell Biology and Development, Institute of Biomeical Science of University of São Paulo (USP), São Paulo, Brazil

**Keywords:** Metabolism, Disease prevention, Quality of life

## Abstract

Physical inactivity has emerged as an important cardiometabolic risk factor; however, the beneficial impacts of physical exercise according physical fitness status are still unclear. To analyze the lipoproteins and immune-endocrine response to acute aerobic exercise sessions performed at different intensities according physical fitness status and evaluated the gene expression in monocyte cells. Twelve individuals, divided into Low and High VO_2max,_ performed three randomized acute exercise sessions at low (<60% VO_2max_), moderate (60–75% VO_2max_), and high (>90% VO_2max_) intensities. Blood samples were collected pre, immediately post, and 60 minutes post-exercise to analyze NEFA, triacylglycerol, non-HDL-c, HDL-c, PAI-1, leptin and adiponectin concentrations. Blood samples were collected from another set of twelve individuals for use in monocyte cell cultures to analyze L-CAT, CETP, and AMPK gene expressions. Low VO_2max_ group pre-exercise exhibited higher postprandial leptin and total cholesterol concentrations than High VO_2max_ group (p < 0.05). Exercise performed in high-intensity promoted a decreased leptin and NEFA levels (p < 0.05, for both), but for PAI-1 levels was decreased (p < 0.05) only for the Low VO_2max_ group. Triacylglycerol levels decreased after all exercise sessions (p < 0.05) for both groups, and HDL-c exhibited decrease during moderate-intensity (p < 0.05), but this scenario was attenuated in Low VO_2max_ group. Low VO_2max_ individuals exhibit some metabolic-endocrine disruption, and acute aerobic exercise sessions performed at low, moderate, and high intensities are capable of modulating metabolic-endocrine parameters, mainly at high-intensity, in a physical fitness-dependent way, given that Low VO_2max_ group was more responsive and seem to be able to appropriate more exercise-related benefits.

## Introduction

Sedentary behavior has emerged as an important cardiometabolic risk factor in the world’s population, favoring the development of several illnesses as obesity, dyslipidemia, cardiovascular diseases (CVD), and all-cause mortality^1–3^. Physical inactivity associated with poor nutrition contributes significantly to the installation and development of metabolic diseases as well as metabolic disorders, such as lower high lipoprotein density (HDL-c), and higher low density lipoprotein (LDL-c), triacylglycerol (TAG) concentrations, and fasting glucose^4^.

Other circulating atherogenic proteins/hormones, such as plasminogen inhibitor-1 (PAI-1), may be modulated by higher lipids and glucose concentrations^5,6^, favoring CVD development. A study has suggested a relationship between glycemia and endothelial biomarkers, verifying a positive association between PAI-1 and hyperglycemia, as well as with fasting glucose, impaired glucose tolerance, diabetes mellitus 2, and insulin resistance in Brazilian adults^5^. Besides that, a recent study revealed that individuals with higher PAI-1 concentrations presented 67% higher risk to develop diabetes *mellitus* type 2;^7^ in addition, PAI-1 is associated with the atherosclerosis process and coronary artery disease mediated by increased LDL-c^6^.

Regular practice of exercise training, independently of the exercise training protocol, is widely recommended as an interesting non-pharmacological strategy to treat and prevent cardiometabolic risks in virtue of anti-inflammatory and anti-atherogenic responses^8^. One positive modulation mediated by aerobic exercise training, especially at moderate intensity protocols, is associated with the regulation of the lipid profile, decreasing concentrations of total cholesterol, LDL-c, triacylglycerol, and increasing HDL-c, being this process facilitated by reverse cholesterol transport mediated by enzymatic modulation of lecithin-cholesterol acyltransferase (L-CAT) and cholesteryl ester transfer protein (CETP)^9^.

In risk population (i.e. obese and/or diabetic), an aerobic exercise protocol performed in intensities with greater fat oxidation seems to be to more effective to increase insulin sensitivity and control glucose levels and body composition^10,11^. Also, the lipid metabolism seems to be regulated by exercise training performed in maximal intensities observing reduction in triacylglycerol, serum leptin, glycerol and non-esterified fatty acids (NEFA) concentrations^10,12^.

In addition to moderate exercise intensity, there is growing interest in studies investigating the effects of physical exercise performed at high intensity, given that this protocol appears to be capable of imposing positive modulation on the metabolism performed in less time, such as by regulating the inflammatory responses in healthy^13^ and overweight/obese men^14^. Hence, the exercise intensity looks as an interesting factor to provide positive responses to metabolism though the impact of acute aerobic exercise performed at different intensities on modeling atherogenic and anti-atherogenic parameters according to physical fitness status is unclear. Since the exercise intensity and physical fitness status influence inflammatory responses and, therefore, we hypothesized that physical fitness status would be a crucial factor to impose the anti-atherogenic role of acute aerobic exercise and individuals with lower physical fitness would be more responsive and get higher modulations mediated by exercise training independently of intensity. Thus, the purpose of the present study was to analyze the lipoproteins and immune-endocrine response to acute aerobic exercise sessions performed at different intensities according physical fitness status and evaluated the gene expression in monocyte cells.

## Material and Methods

### Participant recruitment

A total of 24 healthy male individuals were recruited to participate in the present study, divided into two experiments in order to investigate firstly the peripheral responses modulated by acute exercise sessions (n = 12) and, secondly, the possible molecular mechanisms associated with physical fitness status (n = 12). Thus, in the first experiment, a sample of 12 apparently healthy male individuals (age = 29.7 ± 5.9 years; body mass = 71.7 ± 9.8 kg; body mass index (BMI) = 24.0 ± 2.6 kg/m²) was included and divided according maximal oxygen uptake into Low (VO_2max_ = 41.0 ± 4.2 mL.kg^−1^.min^−1^) and High (VO_2max_ = 62.8 ± 4.9 mL.kg^−1^.min^−1^) VO_2max_ groups based on previous studies conducted by our group^13,15^. All participants were required to complete three acute aerobic exercise sessions, one each at low, moderate, and high intensities. Posteriorly, the other 12 healthy male volunteers with similar characteristics (age = 24.8 ± 4.8 years; body mass = 72.9 ± 9.7 kg; BMI = 22.9 ± 2.1 kg/m²), divided according maximal oxygen uptake into Low (VO_2max_ = 35.3 ± 6.4 mL.kg^−1^.min^−1^) and High (VO_2max_ = 63.0 ± 4.2 mL.kg^−1^.min^−1^) VO_2max_ groups, who did not perform the acute aerobic sessions, were recruited to conduct only the molecular experiments (cell cultures and *real time* PCR analysis). The study subjects were apparently healthy men, without any health disorders that would prohibit physical exercise (mainly at high intensity), such as cardiorespiratory and osteoarticular disease, and who had not used any ergogenic substance or medicine for at least six months prior to the study were included. Written informed consent was obtained from all participants. This study was approved by the local research ethics committee of the Sao Paulo State University “Júlio de Mesquita Filho” and duly registered in Brazil Platform (national electronic system created by the Federal Government to systematize the receipt of research projects involving human beings in Ethics Committees throughout the country) (CAAE: 31168714.6.0000.5402) and the research was conducted according to the 2013 Revision of the Declaration of Helsinki.

### Maximal incremental test and acute aerobic exercise sessions

All the participants (n = 24) performed in the morning, between 8 AM and 12 PM, a maximal incremental test on a cycle ergometer to determine physical fitness status, performance predictors (maximum workload (W_max_), maximal oxygen uptake (VO_2max_), and ventilatory thresholds (aerobic and anaerobic thresholds)). Posteriorly, twelve individuals performed three acute aerobic sessions at low (<60% VO_2max_ - below of aerobic threshold), moderate (60–75% VO_2max_ – between aerobic and anaerobic thresholds), and high (>90% VO_2max_ – above anaerobic thresholds) intensities up to 60 minutes or until voluntary exhaustion. The maximal incremental test and acute aerobic exercise sessions were conducted as previously published by the authors^13,15^.

### Samples collection

Blood sample collection were performed at three times: (I) pre-exercise (1.5 h after breakfast and immediately before to start the exercise sessions), (II) post-exercise (immediately after the end of the exercise sessions), and (III) 60 min post-sessions (passive recovery from physical exercise). Sample processing and treatment were conducted as previously and described by the authors^13^. The dietary compounds offered in the standard breakfast consisted of yogurt, toast and cottage cheese, which are typically consumed by the subjects, respecting the 25% of total energy value, which comprised on average 604 kcal, and the recommended macronutrients proportion of carbohydrates, lipids and protein (≈50, 35 and 15%, respectively).

### Hematocrit analyses

For each blood collection was obtained 100 μL of plasma sample aliquoted in two capillaries (50 μL each), processed for 5 minutes at 12.600 × *g* in a capillary centrifuge (MH 11.5i INBRAS, Brazil) for separates the hematocrit and the measurements were determined by the Van Beaumont equation^16^ where the percentage of plasmatic volume is changed by time-course.

### Biochemical analysis

Triacylglycerol (TAG), cholesterol and fractions (TC, HDL-c, LDL-c) were analyzed by commercial colorimetric kits (Labtest, Brazil) and Non-HDL was calculated by subtraction between Triacylglycerol and HDL-c concentrations. The colorimetric method also was used to determine the non-esterified fatty acids concentrations (NEFA) using a commercial kit (Wako, Japan). Inflammatory parameters (PAI-1, leptin and adiponectin) were analysed by ELISA, according to the manufacturer’s instructions, using commercial kits (R&D System, USA). Intra and inter-assay coefficients of variation (CV) were between 4.4–7.8/6.1–9.5 (PAI-1), 3.0–3.3/3.5–5.4 (leptin) and 2.5–4.7/5.8–6.9 (adiponectin), respectively.

### Peripheral mononuclear cell isolation and monocyte culture

In a fasting state, approximately 20 ml of whole blood of the other twelve individuals were collected in tubes containing EDTA and the samples were added to Histopaque-1077 (Sigma-Aldrich Co. LLC) (1:1) for peripheral blood mononuclear cell (PBMC) isolation, centrifuged at 400 × g for 30 minutes at room temperature. Once isolated, the PBMC was washed with PBS (phosphate buffered saline) and resuspended in 1 mL of enriched-medium RPMI. For separation of monocytes from lymphocytes, 1 × 10^6^ PBMC/mL were incubated for two hours at 37 °C and 5% CO_2_ in cell culture medium (RPMI-1640 Sigma-Aldrich Co. LLC) enriched with glutamine ([2 mM]), sodium bicarbonate ([24 mM]), HEPES ([20 mM]), 10% fetal bovine serum, and antibiotics ([10,000 U/mL] penicillin and [10,000 U/mL] streptomycin) in a 12-well plate (Kasvi - PR/Brazil). In this procedure the monocytes adhere to the bottom of the plate while the lymphocytes remain suspended in the culture medium. Monocyte cells were treated without (control) and with Rosiglitazone (agonist of anti-inflammatory responses mediated by PPAR activation) ([1 μM]) for 24 hours at 37 °C and 5% CO_2_ in wells with 1 mL of final volume. After 24 hours, the adhered cells were harvested with Brazol for gene expression analysis by real-time PCR.

### Quantitative RT-PCR

In this study the RT-PCR was conducted with resting samples in order to analyze the molecular profile and biological machinery, according to physical fitness status, which could be governed, at least in parts, the peripheral alterations associated to exercise training. Total RNA was extracted from monocyte cells with Brazol and cDNA was obtained by commercial kits of High Capacity cDNA Reverse Transcription (Applied Biosystems – Thermo Fisher Scientific) and stored at −80 °C until analysis. L-CAT, CETP and AMPK was measured by RT-PCR using SYBR Green under pre-determined cycling conditions: two steps of 50 °C for 2 minutes and 95 °C for 10 minutes; forty cycles for the amplification: 15 seconds at 95 °C for denaturation, and 60 seconds at 60 °C for annealing and extension at 72 °C for 2 minutes. Table 1 shows the forward and reverse primers. GAPDH was used as constitutive (control). The 2^−ΔΔCT^ equation was performed to determine the relative expression of genes.

**Table 1:** Sequences of forward and reverse primers used for RT-PCR.

Gene	Primer Forward	Primer Reverse
*GAPDH*	ACAACTTTGGTATCGTGGAAGG	GCCATCACGCCACAGTTTC
*L-CAT*	AAGCTGGACAAACCAGATGTG	TAGACAACCCTGGTGTTATCG
*CETP*	AAATCTTCCAAGAGGTTGTCGG	CCATCACTGAAGAATTGACCAC
*AMPK*	GGCACGCATACCCTTGAT	TCTTCCTTCGTACACGCAAATAA

GAPDH = Glyceraldehyde 3‐phosphate dehydrogenase gene; L-CAT = Lecithin-cholesterol acyltransferase; CETP = Cholesteryl ester transfer protein; AMPK = AMP-activated protein kinase.

### Statistical analysis

Firstly, using an effect size for leptin (partial eta squared = 0.32), an alpha value of 5% and 99% power to our sample size was found by analyzing the difference between groups. The Shapiro-Wilk test was used to analyze the sample distribution and the descriptive statistics were composed of means and standard deviations. Independent sample t-tests were applied to compare performance, and inflammatory and metabolic parameters at baseline according to physical fitness status (Low and High VO_2max_). Next, the estimated sphericity was verified using Mauchly’s W test and the Greenhouse-Geisser correction when necessary. To verify differences between intensities, according to physical fitness status (Low and High VO_2max_) in the inflammatory and lipid profile responses, a mixed factorial repeated measures analysis of variance (RMANOVA) was used, where the physical fitness status (Low and High VO_2max_) was included as the between-subject factor (group), and different intensities of exercise (low, moderate, and high intensities) and time (pre-exercise, post-exercise and 60 min post-exercise) were used as the within-subject factors, with the Bonferroni adjustment for multiple comparisons. When a significant interaction was found, Bonferroni’s post hoc was performed and Independent sample t-tests were used to identify the differences between groups or one-way ANOVA to identify the differences between intensities. The comparison in the relative gene expression, according to different treatments (control and Rosiglitazone) in the Low and High VO_2max_ groups was conducted by a mixed factorial RMANOVA, where the physical fitness status (low and high VO_2max_) was included as the between-subject factor (group) and different treatments (control and Rosiglitazone) were used as the within-subject factors with the Bonferroni adjustment for multiple comparisons. If a significant interaction was found, the Bonferroni’s post hoc was performed and Independent sample t-tests were used to identify the differences between groups or time. Effect sizes for the RMANOVA were calculated using partial eta squared (*η*2) for group, time, and interaction. Statistical significance was set at p < 0.05 and the data were analyzed using the Statistical Package for Social Sciences 22.0 (SPSS Inc. Chicago. IL.USA).

## Results

### General modulations mediated by effort

Table 2 shows the general characteristics of the sample, according to physical fitness status (Low and High VO_2max_), performance, inflammatory and metabolic parameters. As expected, there were differences in the oxygen uptake and maximal power obtained in the maximal incremental test between the physical fitness groups. In addition, differences were observed in leptin and total cholesterol postprandial concentrations, and a trend for LDL-c, between low and high VO_2max_ groups, with higher values of both parameters observed in the group with lower physical fitness.

**Table 2:** Performance, inflammatory and metabolic parameters according physical fitness status (Low and High VO_2max_).

Fitness	Low VO_2max_ (n=6)	High VO_2max_ (n=6)	p-value
VO_2max_ (ml.kg.min^−1^)	41.0 ± 4.2	62.8 ± 4.9	<0.001
Maximal Workload (watts)	167.0 ± 33.8	263.4 ± 28.2	<0.001
Leptin (ng.mL^1^)	8.5 ± 5.4	2.7 ± 3.0	0.045
Adiponectin (μg.mL^−1^)	11.2 ± 3.4	12.1 ± 8.0	0.817
PAI-1 (ng.mL^−1^)	20.9 ± 16.7	23.6 ± 23.9	0.822
NEFA (mEq.L)	0.89 ± 0.02	0.91 ± 0.03	0.369
TAG (mg.dL^−1^)	128.6 ± 28.7	150.5 ± 31.7	0.239
TC (mg.dL^−1^)	192.8 ± 24.2	163.3 ± 17.3	0.035
LDL-c (mg.dL^−1^)	122.9 ± 25.0	96.5 ± 16.8	0.057
HDL-c (mg.dL^−1^)	44.1 ± 8.8	36.6 ± 4.2	0.091
Non-HDL (mg.dL^−1^)	148.7 ± 27.2	126.6 ± 14.7	0.111

PAI-1 = plasminogen inhibitor-1; NEFA = Non-esterified fatty acids; TAG = triacylglycerol; TC = total cholesterol; LDL-c = low density lipoprotein; HDL-c = high density lipoprotein.

### Inflammatory responses

**Figure 1:**
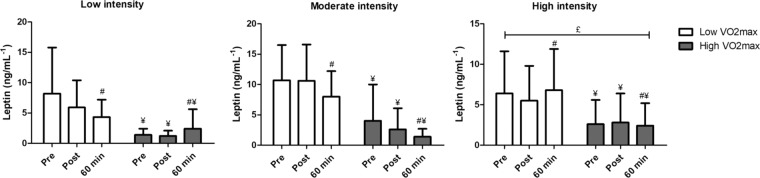
Leptin modulation before exercise sessions performed at low, moderate and high intensities according physical fitness status. ^#^Main effect of time with significant difference from Post; ^£^significant difference between moderate-intensity; ^¥^significant difference between group (High vs Low VO_2max_).

Table 3 presents the comparisons between different intensities according to VO_2max_ on the inflammatory response. For leptin, there was a main effect of time (*F* = 6.480, *p* = 0.007, *η*2 = 0.39) with a decrease after 60 min in relation to post-exercise and a significant difference between intensities (*F* = 3.509, *p* = 0.049, *η*2 = 0.26), with lower values at high-intensity compared to moderate-intensity (p = 0.048). A statistically significant difference was also observed between groups (F = 5.555, p = 0.040, *η*2 = 0.36), but the test of within-subject contrasts showed a tendency to a significant group × intensity × time interaction (*F* = 4.712, *p* *=* 0.055, *η*2 = 0.32) (Fig. 1).

**Table 3:** Comparison between different intensities on the inflammatory response, according physical fitness status (low and high VO_2max_).

Variables	Intensity	Low VO_2max_			High VO_2max_		
		Pre	Post	60 min	Pre	Post	60 min
Leptin (ng.mL^1^)	**Low**	8.2 ± 7.6	5.9 ± 4.5	4.3 ± 2.9^#^	1.4 ± 1.0^¥^	1.2 ± 0.9^¥^	2.4 ± 3.2^¥,#^
	**Moderate**	10.7 ± 5.8	10.6 ± 6.0	8.0 ± 4.2^#^	4.0 ± 6.0^¥^	2.6 ± 3.5^¥^	1.4 ± 1.3^¥,#^
	**High**^**£**^	6.4 ± 5.2	5.5 ± 4.3	6.8 ± 5.1^#^	2.6 ± 3.0^¥^	2.8 ± 3.6^¥^	2.4 ± 2.8^¥,#^
Adiponectin (μg.mL^−1^)	**Low**	10.9 ± 4.9	10.3 ± 3.3	10.5 ± 7.5	14.6 ± 7.5	13.9 ± 8.0	9.4 ± 4.5
	**Moderate**	12.0 ± 2.7	11.4 ± 4.8	11.1 ± 5.3	12.3 ± 8.7	11.5 ± 7.6	12.1 ± 9.6
	**High**	10.7 ± 4.4	12.3 ± 7.3	12.5 ± 6.1	9.3 ± 8.7	8.8 ± 3.9	8.4 ± 6.1
PAI-1 (ng.mL^−1^)	**Low**	20.7 ± 17.4	20.8 ± 15.2	15.2 ± 11.0^b^	26.1 ± 23.7	24.0 ± 23.2	23.7 ± 23.6
	**Moderate**	18.6 ± 14.2	24.1 ± 16.6	14.9 ± 13.1^b^	25.2 ± 24.5	27.1 ± 25.7	28.9 ± 22.2
	**High**	23.3 ± 19.7	33.2 ± 19.7	21.3 ± 19.6^b^	19.6 ± 24.9	20.6 ± 25.2	23.2 ± 24.7
NEFA (mEq.L)	**Low**	0.89 ± 0.03	0.86 ± 0.04	0.90 ± 0.05^b^	0.91 ± 0.04	0.88 ± 0.06	0.98 ± 0.10^b^
	**Moderate**	0.91 ± 0.02	0.91 ± 0.04	0.92 ± 0.02	0.93 ± 0.05	0.90 ± 0.08	0.89 ± 0.02
	**High**^**£**^	0.89 ± 0.05	0.84 ± 0.03	0.93 ± 0.05	0.90 ± 0.02	0.85 ± 0.0^a^	0.94 ± 0.03^b^

PAI-1 = plasminogen activator inhibitor-1; NEFA = Non-esterified fatty acids; ^a^Bonferroni’s post hoc with p < 0.05 compared to rest; ^b^Bonferroni’s post hoc with p < 0.05 compared to post-exercise; ^#^main effect of time with significant difference from Post; ^£^significant difference between moderate-intensity; ^¥^significant difference between group (High vs low VO_2max_).

For adiponectin, there was a significant group × intensity interaction (*F* = 4.068, *p* = 0.033, *η*2 = 0.29). The Bonferroni’s post hoc showed a tendency to lower concentrations at high-intensity in relation to low-intensity (p = 0.056) only for the high VO_2max_ group, but without significant difference between groups (*F* = 0.002, *p* = 0.964, *η*2 = 0.001) or group × intensity × time interaction (*F* = 0.609, *p* = 0.659, *η*2 = 0.06).

For PAI-1, there was a significant group × time interaction (*F* = 5.014, *p* = 0.017, *η*2 = 0.33). The Bonferroni’s post hoc showed a significant decrease only for the low VO_2max_ group after 60 min in relation to post-exercise (p = 0.013). A significant group × intensity interaction (*F* = 8.904, *p* = 0.002, *η*2 *=* 0.47) was observed. Despite a lower concentration at low (p = 0.073) and moderate (p = 0.074) intensities compared to high-intensity, the post hoc did not identify significant differences between intensities and there were no significant differences between groups (*F* = 0.064, p = 0.805, *η*2 *=* 0.006) or group × intensity × time interactions (*F* = 0.743, *p* = 0.569, *η*2 = 0.07).

For non-esterified fatty acids (NEFA) there was a significant intensity × time interaction (*F* = 4.756, p = 0.003, *η*2 = 0.32). The Bonferroni’s post hoc showed statistically significant differences between moderate and high-intensities post-exercise (p = 0.027). Furthermore, for low-intensity, there was a significant increase after 60 min in relation to post-exercise (p = 0.005) and for high-intensity, there was a significant decrease post-exercise in relation to rest (p = 0.020), but an increase after 60 min compared to post-exercise (p < 0.001).

### Lipid responses

Table 4 shows the comparison between different intensities according to the VO_2max_ in the lipid profile. For triacylglycerol, there was a main effect of time (*F* = 10.724, *p* *=* 0.001, *η*2 = 0.52) with lower values post-exercise and after 60 min in relation to rest (p < 0.05). However, there were no significant group × intensity × time interactions (*F* = 1.091, *p* = 0.374, *η*2 = 0.10) or significant differences between groups (*F* = 0.940, *p* = 0.355, *η*2 = 0.09).

**Table 4:** Comparison between different intensities, according physical fitness status (low and high VO_2max_) in the lipid profile.

Variables	Intensity	Low VO_2max_			High VO_2max_		
		Pre	Post	60 min	Pre	Post	60 min
TAG (mg.dL^−1^)	**Low**	123.2 ± 30.1	111.9 ± 23.9^*^	116.3 ± 25.6^*^	151.1 ± 35.1	134.3 ± 30.1^*^	124.9 ± 15.7^*^
	**Moderate**	122.5 ± 16.1	114.3 ± 9.9^*^	109.0 ± 4.8^*^	151.1 ± 41.3	118.6 ± 21.3^*^	112.3 ± 6.5^*^
	**High**	140.1 ± 52.9	131.9 ± 39.5^*^	133.1 ± 37.6^*^	149.3 ± 29.9	141.5 ± 26.7^*^	132.9 ± 14.3^*^
TC (mg.dL^−1^)	**Low**	186.8 ± 27.1	181.7 ± 27.7	180.6 ± 37.5	166.5 ± 23.4	184.4 ± 34.6	182.8 ±± 31.7
	**Moderate**	194.4 ± 31.2	177.1 ± 25.3	170.2 ± 20.0	151.7 ± 11.9	160.7 ± 17.2	155.5 ± 14.8
	**High**	197.2 ± 31.3	186.9 ± 34.0	190.8 ± 29.7	171.7 ± 20.7	166.6 ± 15.6	172.6 ± 14.3
LDL-c (mg.dL^−1^)	**Low**	118.7 ±± 27.4	117.0 ± 26.6	112.2 ± 35.1	98.6 ± 20.7	121.6 ± 30.0	118.2 ± 28.1
	**Moderate**	123.6 ± 36.0	107.4 ± 25.7	100.0 ± 22.9	86.6 ± 14.0	101.5 ± 14.6	96.5 ± 18.0
	**High**	126.7 ± 26.7	119.0 ± 36.2	122.1 ± 36.9	104.4 ± 19.0	101.9 ± 13.1	106.2 ± 13.9
HDL-c (mg.dL^−1^)	**Low**	43.4 ± 11.0	42.3 ± 9.5	45.2 ± 10.0	37.7 ± 5.9	36.0 ± 5.4	39.6 ± 9.3
	**Moderate**	46.4 ± 7.3	46.8 ± 7.9	48.3 ± 6.6	34.8 ± 3.3^¥^	35.4 ± 4.0^¥^	36.5 ± 4.6^¥^
	**High**	42.5 ± 10.8	41.6 ± 10.3	42.1 ± 15.1	37.4 ± 4.6	36.4 ± 3.0	39.8 ± 4.2
Non-HDL (mg.dL^−1^)	**Low**	143.3 ± 28.5	139.3 ± 25.3	135.4 ± 35.7	128.8 ± 20.3	148.5 ± 33.9	143.2 ± 29.7
	**Moderate**	148.1 ± 35.5	130.3 ± 26.5	121.8 ± 22.2	116.8 ± 11.4	125.2 ± 18.3	119.0 ± 18.9
	**High**	154.7 ± 34.2	145.4 ± 33.5	148.7 ± 38.4	134.3 ± 17.2	130.2 ± 16.8	132.8 ± 16.0

TAG = triacylglycerol; TC = total cholesterol; LDL-c = low density lipoprotein; HDL-c = high density lipoprotein. *Main effect of time with significant difference from rest; ^¥^significant difference between group (High vs low VO_2max_).

For total cholesterol, there was no significant group × intensity × time interaction (*F* = 0.611, *p* = 0.657, *η*2 = 0.06) or significant differences between groups (*F* = 2.482, *p* = 0.146, *η*2 = 0.20). When analyzing the LDL-c, there was a group × time interaction (*F* = 3.595, *p* = 0.046, *η*2 = 0.26) with a tendency to a significant difference between the groups at rest (p = 0.057) though no group × intensity × time interactions (*F* = 0.908, *p* = 0.468, *η*2 = 0.08) or differences between groups were observed (*F* = 1.268, *p* = 0.286, *η*2 = 0.11). For HDL-c, there was a group × intensity interaction (*F* = 4.236, *p* = 0.029, *η*2 = 0.30). The Bonferroni’s post hoc identified a significant difference between groups at moderate-intensity (p = 0.006), while for Non-HDL, there was no group × intensity × time interaction (*F* = 0.658, *p* = 0.625, *η*2 = 0.06) or differences between groups (*F* = 0.736, *p* = 0.411, *η*2 = 0.07).

### Gene expression by RT-PCR analyzes

**Figure 2:**
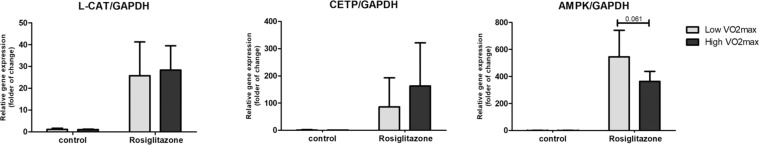
Comparison of the relative gene expression, according to control and Rosiglitazone treatment in the low and high VO_2max_ groups.

Figure 2 presents the relative gene expression, according to control and Rosiglitazone treatment in the low (VO_2max_ = 35.3 ± 6.4 ml.kg.min^−1^) and high (VO_2max_ = 63.0 ± 4.2 ml.kg.min^−1^) VO_2max_ groups. There were statistically significant increases in L-CAT (*F* = 43.717, *p* *<* 0.001, *η*2 = 0.81), CETP (*F* = 10.069, *p* = 0.010, *η*2 = 0.50), and AMPK (*F* = 111.307, *p* < 0.001, *η*2 = 0.92) for Rosiglitazone treatment compared to control, but there were no significant differences between low and high VO_2max_ groups, despite a tendency to greater AMPK in the Low VO_2_ group (p = 0.061) when Rosiglitazone was used.

## Discussion

The main findings of the present study were (I) leptin levels exhibited a decrease after aerobic exercise sessions, mainly at high-intensity for both groups (high VO_2max_ group showed lower concentrations at all moments when compared to the low VO_2max_ group); (II) PAI-1 levels showed a decrease after all exercise intensity sessions in post-exercise than pre-exercise period only for Low VO_2max_ group; (III) NEFA levels showed a decrease post-exercise than rest; (IV) For lipid profile, triacylglycerol levels decreased post-exercise for all exercise sessions than rest, and HDL-c levels showed a difference between groups at moderate intensity (with lower values in the high VO_2max_ group). These findings lead us to understand that acute aerobic exercise is able to impose an anti-atherogenic profile in a physical fitness-dependent way.

Non-health condition associated with poor physical fitness is extensively debated, given that sedentary individuals exhibit augmented fat body and alterations in the metabolic parameters, such as total cholesterol, HDL-c, LDL-c, insulin, and leptin^17^. Higher leptin concentration is positively associated with sedentary behavior, but this relationship may be attenuated breaking the sedentary time by exercise physical practice^18^. On the other hand, trained individuals sowed lower levels of TAG, fasting insulin, and leptin, parallel with higher levels of HDL-c and these parameters are modified by age, sex, and health status^19^. These data explain the difference between physical fitness range, mainly for leptin and total cholesterol concentrations at baseline despite postprandial measurement.

Leptin acts as a signal informing the central nervous system about the ingested and stored energy in the fat body. This is recognized as an energy sensor with an important function in energy balance, favoring energy conservation and decreased thermogenesis^20^. Based on this scenario, declines after exercise sessions may be marked by alterations in energy availability, given that higher energy expenditure leads to greater bioavailability of the energy substrate to maintain the contractile activity.

Gomez-Merino and colleagues^21^ observed a decrease in serum leptin after prolonged physical activity (5 days of combat course walking distances of 25–35 km each night) performed by healthy men (VO_2max_ = ~3.74 L.min^−1^) and suggest the increase in circulating free fatty acids as a sensor of leptin level decrease. Similar results were observed by Duclos and colleagues^22^ after 2 hours of running performed by male runners (VO_2max_ = 62.3 ± 1.8 ml.kg.min^−1^) and a negative correlation was found between free fatty acid and leptin levels. In our data, we also found an inverse correlation between the same variables immediately post-exercise (r = −0.622; p = 0.031), independently of physical fitness status, but only at high-intensity. Thus, our results are in agreement with these studies given that after an acute aerobic exercise sessions, was found a decrease in leptin concentration parallel with increased non-esterified fatty acids, evidencing metabolic regulation which may reflect enhanced lipolysis from adipose tissue and an increased use of fatty acids for energy during exercise. Besides energetic modulation, the anti-atherogenic effects of exercise training practice are well documented in the literature, with impacts on lipid profile and atherogenic biomarker regulation, such as PAI-1. Lira and colleagues^23^ suggested that PAI-1 concentration at rest is linked to physical fitness status, given that lower concentrations are verified in highly-trained individuals, with high training volume, when compared with sedentary individuals. It is important to highlight that PAI-1 levels are considered an atherogenic parameter with an important contribution to the balance of fibrinolytic processes, inhibiting the action of tissue plasminogen activator^24^.

When analyzing low and high VO_2max_ groups we verified, although not significant, an increase in PAI-1 concentrations after the acute aerobic exercise sessions; however, these values were significantly reduced 60 minutes after the end of the effort and these changes were observed only in the low VO_2max_ group. A previous study conducted by our group observed a significant increase in PAI-1 concentrations immediately after the exercise session at moderate intensity (70% of speed associated with VO_2peak_)^25^, as well as a study by Buchan and colleagues^26^, and it seems that submaximal exercises (at 90% of individual anaerobic threshold for 60 min) are also able to increase PAI-1 concentrations, although not significant, in healthy men, returning to lower values later, suggesting a transient coagulation state associated with physical exercise.

Studies evidence the cardioprotective effects of regular practice of physical exercise in several populations. Jahangard and colleagues^27^ showed a reduction in the antigen and activity of PAI-1 in sedentary healthy women after 10 sessions of submaximal aerobic cycling (3 times per week at 70% of maximal heart rate during 25 minutes, 3 times per week); in addition, similar effects were observed in healthy men after 6 months of endurance training (3 times per week at 85% of heart rate reserve during 45 minutes) decreasing PAI-1 activity by 37%^28^.

In the same line, when comparing inactive, physically active, and highly (competitive runners) active men, at rest, the highest PAI-1 activity was observed in the inactive men and the lowest values in the highly active group; however, the PAI-1 activity decreased with acute exercise (maximal graded treadmill exercise)^29^ and the authors suggested that these modulations may be mediated by lipid-related changes associated with exercise, given that a previous study found correlations between PAI-1 and triacylglycerol^30^. These findings corroborate our results and explain, at least in part, the positive response of physical exercise mainly for individuals with a low physical fitness status, given that we found PAI-1 and triacylglycerol reductions even after a single exercise session. Another important metabolic pathway that could regulate lipid abnormalities is the reverse transport of cholesterol, primarily mediated by CETP and L-CAT enzymes, but improvement in activation of this mechanism cannot be fully considered as we did not observe any difference in the gene expression of these enzymes.

In this perspective, is well documented that regular exercise training is capable to improve several metabolic pathways and lipids-related mechanisms as reverse cholesterol transport that is responsible for regulating lipids in the tissues and circulating. HDL-c plays a critical role in cholesterol efflux removing cholesterol from peripheral cell membranes for later transport to the liver for excretion, and in this process, cholesterol efflux from cells to HDL-c does not occur at the plasma level but rather in the subendothelial layers of the arteries^31,32^. This mechanism may explain, at least in parts, the lower HDL-c values in the High VO_2max_ group, when compared with Low VO_2max_ group, suggesting a possible efficiency in the efflux cholesterol enhanced by fitness status Since the decrease in circulating lipoprotein concentrations may not reflect a deficiency in HDL-c synthesis, but an increased removal of the circulating particle to other tissues.

Some limitations should be mentioned about the study. Despite offering a standard breakfast to all individuals in order to equalize the energy intake prior to exercise, a dietary intervention and/or food control were not carried out in the days prior to the physical exercise sessions and may be a confounding factor. In addition, another limiting factor is the use of two distinct groups for the peripheral and molecular analyzes even though taking care to include similar pairs in the two phases of the study. Future studies should be conducted with the same subjects controlling nutrition aspects, as well as with samples of other groups (e.g., women, older individuals, and risk populations) in order to verify the positive effects mediated by aerobic exercise, independently of intensity, considering the physical training specificities targeted to specific groups. Furthermore, investigation of other proteins and regulatory molecules which participate directly in modulating the anti-atherogenic response need to be included in future studies, and can be highlighted as a limitation of this study.

Taken together, our data show that acute aerobic exercise sessions performed at low, moderate, and high intensities are able to modulate atherogenic and anti-atherogenic parameters, mainly performed at high-intensity, and in a physical fitness-dependent way, given that Low VO_2max_ group was more responsive and seem to be able to appropriate more exercise-related benefits.
